# Inclusive Healthy Aging in a Global Society: Strengthening Community and Cognitive Vitality

**DOI:** 10.1093/geroni/igaf122.302

**Published:** 2025-12-31

**Authors:** Su-I Hou

## Abstract

This symposium explores global perspectives on aging, focusing on societal perceptions, loneliness, cognitive decline prevention, and psychological well-being. Through multidisciplinary and international viewpoints, it highlights research-driven strategies for fostering inclusive communities and promoting cognitive and emotional health among older adults worldwide. Prof. Hou (University of Central Florida) will compare older adults in neighborhood lunch programs (NLP) and lifelong learning programs (LLP) in the U.S., examining how community engagement shapes perceptions of aging, financial security, and political influence. Drs. Pan and Liu (Vrije Universiteit Brussel & National Cheng Kung University) will explore social and emotional loneliness among community-dwelling older adults in Taiwan during the early pandemic. Their findings on community and individual risk factors will inform culturally adaptive intervention strategies. Drs. Chou and Hou (UCF) will analyze the role of polypharmacy in moderating the relationship between instrumental activities of daily living (IADL) and physio-cognitive decline syndromes (PCDS), emphasizing the need for medication management in supporting cognitive and physical health. Ms. Perera (FINGERS Brain Health Institute) will present updates from the MET-FINGER study, a European-led trial combining lifestyle interventions with metformin to prevent cognitive impairment. This study highlights the potential for precision-prevention strategies in reducing dementia risk globally. Ms. Chang (University of Rochester) will examine the impact of vision loss on psychological well-being among older adults in the U.S., offering insights into interventions applicable across diverse healthcare systems. Together, these studies provide global insights into aging, offering evidence-based strategies to enhance community engagement, cognitive resilience, and psychological well-being in later life. This is a collaborative symposium between the Aging Among Asians and International Comparisons of Healthy Aging Interest Groups.

